# Magic Curiosity Arousing Tricks (MagicCATs): A novel stimulus collection to induce epistemic emotions

**DOI:** 10.3758/s13428-020-01431-2

**Published:** 2020-07-10

**Authors:** Hiroki Ozono, Asuka Komiya, Kei Kuratomi, Aya Hatano, Greta Fastrich, Jasmine April Louise Raw, Anthony Haffey, Stefanie Meliss, Johnny King L. Lau, Kou Murayama

**Affiliations:** 1 Faculty of Law, Economics and Humanities, Kagoshima University, Korimoto, Kagoshima, 890-0065 Japan; 2 Graduate School of Humanities and Social Sciences, Hiroshima University, Higashi-Hiroshima, Japan; 3 Faculty of Psychology, Aichi Shukutoku University, Nagakute, Japan; 4 Department of Cognitive Psychology in Education, Graduate School of Education, Kyoto University, Kyoto, Japan; 5 Research Institute, Kochi University of Technology, Kochi, Japan; 6 School of Psychology, University of Western Australia, Perth, Australia; 7 School of Psychology and Clinical Language Sciences, University of Reading, Earley Gate, Whiteknights, Reading, RG6 6AL UK

**Keywords:** intrinsic motivation, epistemic emotions, curiosity, science of magic

## Abstract

There has been considerable interest in empirical research on epistemic emotions, i.e., emotions related to knowledge-generating qualities of cognitive tasks and activities such as curiosity, interest, and surprise. One big challenge when studying epistemic emotions is systematically inducting these emotions in restricted experimental settings. The current study created a novel stimulus set called Magic Curiosity Arousing Tricks (MagicCATs): a collection of 166 short magic trick video clips that aim to induce a variety of epistemic emotions. MagicCATs are freely available for research and can be used in a variety of ways to examine epistemic emotions. Rating data also support that the magic tricks elicit a variety of epistemic emotions with sufficient inter-stimulus variability, demonstrating good psychometric properties for their use in psychological experiments.

Recent years have seen a surge of interest in the topic of so-called epistemic emotions. Epistemic emotions refer to a group of emotions which are related to the knowledge-generating qualities of cognitive tasks and activities (Brun, Doğuoğlu, & Kuenzle, 2008; Muis, Chevrier, & Singh, 2018). These emotions typically include surprise, curiosity and interest. Recent studies have revealed that these epistemic emotions have profound implications for cognitive processing and learning. Surprise is caused by the discrepancy between expected and actual outcomes, and this discrepancy (which can be described as ‘prediction error’) is the basis of learning and decision-making (Dole & Sinatra, 1998; Rescorla & Wagner, 1972). Stahl and Feigenson (2015) showed that even 11-month-old infants can learn from events better when their expectation was violated (i.e., they were surprised). A number of recent studies have also found that the strength of the feeling of curiosity or interest triggered by the presentation of trivia questions predicts memory accuracy of the answers to the questions (Fastrich, Kerr, Castel, & Murayama, 2017; Kang et al., 2009; Marvin & Shohamy, 2016; McGillivray, Murayama, & Castel, 2015; Wade & Kidd, 2019) as well as that of irrelevant materials that were incidentally presented (Galli et al., 2018; Gruber, Gelman, & Ranganath, 2014).

One of the challenges of research on epistemic emotions is that it is not easy to induce these epistemic emotions in experimental settings. In controlled experiments, including those with neuroscientific facilities such as functional magnetic resonance imaging (fMRI), researchers need a number of short repeated trials to ensure the reliability of the task. However, triggering epistemic emotions in such a short time frame is challenging because epistemic emotions, by definition, require people to cognitively process the task; epistemic emotions are not something that can be immediately triggered upon presenting a stimulus. In addition, even if it is possible to induce epistemic emotions in a short time frame, the magnitude of the emotion is likely to be insufficient to cause a psychological response and/or behavioral change. Thus, there are limited experimental materials that claim to induce epistemic emotions. In fact, the vast majority of studies in experimental psychology use trivia questions or similar knowledge questions to induce epistemic emotions, particularly curiosity (Baranes, Oudeyer, & Gottlieb, 2015; Kang et al., 2009; Litman, Hutchins, & Russon, 2005; Metcalfe, Schwartz, & Bloom, 2017; Murayama & Kuhbandner, 2011)^1^.

In the current study, we have validated a stimulus set called Magic Curiosity Arousing Tricks (MagicCATs): a collection of 166 novel short magic trick video clips that trigger people's epistemic emotions in experimental settings. The videos of MagicCATs are available for researchers (see "Stimulus Availability" section in Methods) and the current article reports basic characteristics and the norms of these magic trick video clips. There are already several studies that have used magic tricks as stimuli to trigger epistemic emotions, including neuroimaging studies (e.g., Parris, Kuhn, Mizon, Benattayallah, & Hodgson, 2009, Danek, Öllinger, Fraps, Grothe, & Flanagin, 2015), but to the best of our knowledge, there are no standardized stimuli that are freely available to researchers. In contrast to other materials, one unique aspect of magic tricks is that they induce a strong sense of violation of expectation and surprise (Danek et al., 2015). Surprise and curiosity/interest are obviously interrelated epistemic emotions (Pekrun, Vogl, Muis, & Sinatra, 2017), but the materials available thus far (e.g., trivia questions) are not designed to induce surprise to evoke curiosity (for an exception, see Vogl, Pekrun, Murayama, & Loderer, 2019; Vogl, Pekrun, Murayama, Loderer, & Schubert, 2019). Perhaps as a consequence of this curiosity research tends to focus on uncertainty as a major triggering factor (e.g., van Lieshout, Vandenbroucke, Müller, Cools, & de Lange, 2018), and the role of surprise in relation to curiosity and interest has been relatively under-examined.

Another important feature of magic tricks is their relatively strong, intuitive, and universal appeal. Because magic tricks are intended to create a strong violation of expectation, spectators are naturally motivated to understand why the expectation is violated ("why did this happen?"), which is thus likely to induce relatively strong feelings of epistemic emotions. An advantage of magic tricks is that they mainly consist of nonverbal information such as vanishing or appearing materials. This nonverbal nature of the stimuli makes it easier for people to intuitively understand the content. As a result, magic tricks can trigger epistemic emotions regardless of the participant’s language, educational and cultural backgrounds. Of course, we are not claiming that magic tricks are superior to existing stimuli to trigger epistemic emotions. There are some obvious limitations such as difficulties controlling stimulus length. However, given the unique advantages of magic tricks, we believe that the current stimulus set provides complementary benefits to the researchers studying epistemic emotions.

There are different ways to utilize MagicCATs in research, but one common context is an experiment in which researchers intend to elicit different levels of epistemic emotions on a trial-by-trial basis to examine the within-person correlates of epistemic emotions. We have already conducted one neuroimaging experiment using MagicCATs and validated the effectiveness of the stimuli. In Lau, Ozono, Kuratomi, Komiya, & Murayama (2020), we presented participants with a series of 36 magic trick video clips from MagicCATS to induce feeling of curiosity and asked them to decide whether they would be willing to risk receiving electric shocks to satisfy the curiosity to know the solution of the trick. Self-reported ratings of curiosity for each magic trick was significantly associated with the decision to accept the risk to receive electric shocks on a trial-by-trial basis, indicating that the magic trick videos successfully induced curiosity.

The collection of magic trick video clips we provide would also benefit the recent growing area called the *“science of magic”*, which investigates human cognitive mechanisms using magic tricks (for review, see Kuhn, Amlani, Rensink, 2008; Kuhn, 2019; Thomas, Didierjean, Maquestiaux, & Gygax, 2015). Broadly speaking, magic tricks can be classified by the three general methods used by magicians: misdirection, illusion and forcing (Kuhn et al., 2008). By embedding these types of magic tricks in psychological experiments, we can gain unique insight into cognitive processes. For example, the technique of *misdirection*, i.e., manipulating the spectator away from the cause of magic effect (Kuhn et al., 2008), is useful to investigate mechanisms of attention (e.g., Barnhart & Goldinger, 2014; Kuhn & Findlay, 2010; Kuhn & Land, 2006; Wiseman & Nakano, 2016). Also, many magic tricks are based on visual or cognitive illusions (e.g., Macknik, Martinez-Conde, & Blakeslee, 2010). Investigating how magicians use such *illusions* in practice may lead to new insights in perception and cognition (Ekroll, Sayim, and Wagemans, 2013). Furthermore, some magic tricks *force* the spectators to choose a certain object while the spectators believe that they made the choice out of their free will. Investigating how and why spectators have such false beliefs can lead to better understanding of human free will and agency (Kuhn, Pailhès, & Lan, 2020; Olson, Amlani, & Rensink, 2013; Ozono, 2017; Pailhès, & Kuhn, 2020). Our magic trick videos include misdirection, illusion, and forcing as well as other various trick mechanisms (e.g., tricks utilizing mathematical logic or physical principles), allowing researchers to pursue a variety of psychological research questions.

The current paper describes how we created MagicCATs, which aims to induce epistemic emotions in psychological experiments. We then provide rating data and perform quantitative analysis to examine the psychometric properties of MagicCATs. Specifically, we show that the magic tricks elicit a variety of epistemic emotions (surprise in response to the trick, interest in the trick, and curiosity in the solution) with sufficient inter-stimulus variability.

## Methods

### Creating MagicCATs

Four male magicians, including a champion of an international magic competition, performed 145 magic tricks in total. These performances were filmed in two studios (one studio for one magician and another studio for the other three magicians) by professional photographers using high resolution video cameras. The magicians selected magic tricks that would maximize the variety of materials (e.g., playing cards, coins, sponges etc.) and the types of the tricks (e.g., vanishing, transportation, prediction, etc.). Magicians also ensured that the magic tricks were heterogeneous enough to induce different levels of epistemic emotions (i.e., surprise, interest, and curiosity). As most previous research on curiosity and interest were comparing the responses to the stimuli that induce either a low or high-level of curiosity and interest (Fastrich et al., 2017; Gruber et al., 2014; Kang et al., 2009), it is important to have sufficient inter-stimulus variability (i.e., it is critical to include magic tricks that are relatively less surprising/interesting). See Appendix Table 6 for the details of the MagicCATs.

All videos were then edited using Adobe® Premiere Pro CC® (2015) software to have a similar monotonic (dark) background, size (720 x 404 pixels) and viewing focus. The videos were muted, and English subtitles were added in a few videos, when necessary. The face of the magician was obscured as much as possible to avoid potential distraction due to their appearance and facial expressions. This editing also helps minimize potential responses to the gender of the magicians reported by Gygax, Thomas, Didierjean, & Kuhn (2019). Twenty-one magic tricks out of the 145 tricks were relatively long and included a sequence of more than one tricks. For these videos, we created a short-version focusing on the first trick that was presented, in addition to the original long version, thus giving researchers more flexibility in stimulus selection (e.g., choosing stimuli that fit within a specific time constraint). In total, we created 166 video clips including the 21 long video clips of tricks that are accompanied by 21 short versions and 124 other videos. These videos ranged between 8 and 155 s long (mean = 37.3; median = 31; SD = 23.5). Without including the long versions, the videos raged between 8 and 105 s long (mean = 33.1; median = 30; SD = 19.8) . Here are the URLs of three video samples. https://youtu.be/aVM-7VlXE5k, https://youtu.be/BAJ25HmsF-I, https://youtu.be/ehgSKYxVL3M

### Rating task

#### Participants

A total of 495 participants took part in the rating task through Amazon Mechanical Turk. A total of 470 US participants were paid $3.5 for approximately 35 min of study participation, whist the first 25 participants were paid $2.5 before our re-calculation of the more realistic study completion time. Prior to the main data analysis, we excluded 44 participants who either (a) took more than +2 SD longer than average to complete the experiment (there is no participant who took shorter than 2SD below the average duration); (b) gave identical ratings on more than three questions for all trials; (c) answered "no" to the "clarity of the trick" question for all the presented video clips (see below); (d) indicated some issues in video presentation and internet connection in post-questions; or (e) indicated that they were distracted during the experiment or had already taken part in a similar experiment previously. This exclusion led to the final sample of 451 participants; 259 males and 192 females (mean age = 36.10, SD = 10.34, range = 20–71).

#### Stimulus lists

It was impractical for any single participant to view and rate all the 166 video clips in MagicCATs, particularly as there were some duplicated magic tricks due to the short- and long-version as described above. To address this, we split the 166 video clips into nine lists and only presented each participant video clips from two of the lists. Our design is an adopted version of a Balanced Incomplete Block (BIB) spiraling procedure in the literature of test theory (Fleiss, 1981; Hanani, 1961).

The nine lists (Lists 1–9) were created in the following manner. Lists 1 and 2 consisted of 21 tricks from the short and long versions of the video clips mentioned earlier. Short and long versions of the video clips were assigned evenly to these lists (i.e., List 1 included ten short- and 11 long-version video clips; List 2 included 11 short- and ten long-version video clips). Video clips from the same magic trick were not assigned to the same list, thus when List 1 included a short version of a magic trick, List 2 included a long version of the same magic trick. The other 124 video clips were assigned to the remaining 7 lists (List 3 to List 9), resulting in 17 or 18 video clips for each of the lists. When creating these lists, we attempted to minimize the difference in the total duration of the video clips between lists. Consequently, the total duration of the video clips in Lists 1 and 2 were 16–17 min and 9–10 min in Lists 3 to 9.

#### Procedure

Participants were presented with video clips from two of the nine lists in a randomized order. The assignment of the lists was randomly determined with the constraint that participants were not presented both Lists 1 and 2 because they included video clips from the same magic tricks but of different lengths. For each video clip participants gave five different ratings: (a) whether they understood the intention of the magic trick or not (*clarity* of the trick); (b) how surprised they were at the magic trick (*surprise* in response to the trick); (c) how interesting the magic tricks were (*interest* in the trick); (d) how confident they were that they had figured out the solution to the trick (*confidence* in the solution); and (e) how curious they were about how the magic trick was done (*curiosity* of the solution). Clarity of the trick, which assessed whether participants understand what happened after seeing the video clip, was rated using a binary response scale (Yes/No); we included this rating to be used as an exclusion criterion. The other four questions were rated on 10-point Likert scales ranging from 1 (not at all) to 10 (very much).

At the end of the session, participants gave some post-experiment ratings. First, participants reported how much they were interested in the magic tricks in general (1 being not at all and 10 being very much) and whether they perform magic tricks themselves (1 being not at all, 2 being a little bit and 3 being frequently). Second, they reported any video presentation or internet connection problems that had occurred during the experiment; whether they were doing anything else during the experiment and whether they had already participated in another experiment with the same videos. We emphasized that their compensation would not be affected by their responses. As indicated above, these questions were used to exclude participants from the main data analysis who seemed to have been disengaged during the study.

## Results

Analysis on the post hoc questions showed that participants were generally interested in the magic tricks M = 7.13, SD = 2.05. Only six participants indicated that they frequently performed magic tricks; 379 participants indicated that they had never performed a magic trick. In the following, we did not use the data for Trick 9 because it mistakenly contained sounds (we still include this magic trick in the final stimulus set in Appendix Table 6).

Table 1 reports the descriptive statistics of the main variables (clarity of the trick, surprise in response to the trick, interest in the trick, confidence in the solution, and curiosity in the solution) with video clips as the unit of analysis. On average, participants understood the intention of the magic tricks the majority of the time (86.03%). When looking at the average proportion of participants who understood the magic trick for each video clip (Appendix Table 7), the majority of the video clips (156 out of the 165 video clips) were understood by more than 70% of participants. However, there were nine video clips that were understood by less than 70% of participants. In the following analysis, all the data of the trials in which the intentions of the magic tricks were unclear to participants were removed.

**Table 1:** Descriptive statistics for the 166 magic trick videos

	Clarity of the trick	Surprise in response to the trick	Interest in the trick	Confidence in the solution	Curiosity in the solution
Mean	.86	5.58	5.70	4.14	5.71
SD	.09	0.81	0.77	0.84	0.72
Min	.47	3.86	3.95	2.54	3.87
Max	.97	7.65	7.88	7.23	7.89

Note: The rating scale ranged from 1 to 10. We first computed the mean of each video clip (across participants) and then computed the descriptive statistics. Except for the clarity, we removed the trials that were unclear to participants.

Participants reported a moderate level of surprise in response to the trick, interest in the trick, and curiosity in the solution (M = 5.58, 5.70, and 5.71, SD = 0.81, 0.77, 0.72, respectively, on a 1–10 scale). These average rating values were consistent with the fact that we intended to include both surprising and less surprising magic tricks to ensure the heterogeneity of the stimulus set. The average confidence in the solution is relatively low (M = 4.14). Note that this is a subjective rating of confidence and we do not have objective data to demonstrate that participants indeed correctly guessed the solution behind some of the magic tricks.

To further examine whether our new stimuli can appropriately evaluate within-person variability of epistemic emotions, we applied a mixed-effects model to the data to decompose the three distinct variance components: participant variance, video variance, and participant x video variance. Participant variance represents overall individual differences between participants (i.e., some participants had relatively high experience of surprise compared to others across all of the video clips), whereas video variance represents differences in ratings between video clips (e.g., some video clips are more surprising than other video clips to all participants). Participant x video variance represents individual differences in participants' responses to different video clips (e.g., some participants were interested in a specific video clip whereas others were not). Note that participant x video variance also includes variance from measurement errors, which we cannot statistically separate. In most of the previous literature on epistemic emotions (e.g., Fayn et al., 2019; Fastrich et al., 2017; Vogl et al. 2019, 2019), within-person variability reflected both the stimulus variance and participant x stimulus variance (i.e., video variance and participant x video variance in this study).

The mixed model regression found that the majority of the variance can be explained by the participant x video clip for all the ratings (Table 2). The random effect of participant variance explains about 36–43% of the response variance. The random effect of video is the smallest, explaining about 5–7% of the variance. These results indicate that these materials have sufficient within-person variability (57–64%) to examine intra-individual fluctuation of these epistemic emotions. The findings are also largely consistent with the findings of Fastrich et al. (2017) who used trivia questions.

**Table 2:** Variance components of the ratings

	Participant variance	Video clip variance	Participant x video variance
Surprise in response to the trick	37.9%	7.4%	54.7%
Interest in the trick	37.3%	7.2%	55.5%
Confidence in the solution	36.3%	7.1%	56.6%
Curiosity in the solution	42.8%	4.9%	52.3%

One limitation of the variance decomposition analysis in Table 2 is that we cannot dissociate the participant x video variance from measurement error variance; it is possible that within-person variance that we reported is an overestimation. Although it is difficult to perfectly address the issue, to gain more insights into the rating data we conducted further mixed-effects modeling to further decompose the variance by treating ratings of epistemic emotions as an additional factor. More specifically, we regarded the data as three mode data of participant x videos x type of epistemic emotions (emotion type being either surprise in response to the trick, interest in the trick or curiosity in the solution), and conducted mixed-effects modeling to estimate the variance components of each factor and their interactions. We excluded confidence from the analysis because confidence was not assessed as an epistemic emotion. As emotion type variance was very small and caused a convergence error, we eliminated this term from the final model. Table 3 includes these results. Note that participant x video x emotion type variance is confounded with measurement error variance. One important finding from this analysis is that the large contribution of the participant x video clip observed in the original variance decomposition model (Table 2) is now largely absorbed into participant x video variance (not participant x video x emotion type variance), which is no longer confounded with error terms. This finding means that measurement error was not a major source of the participant x video clip variance observed in the original model. Another important observation is that emotion type explained a relatively small portion (20.1%) of the total variance (Video x emotion type + Participant x emotion type + Participant x video x emotion type, which includes measurement errors). These results suggest the possibility that participants did not make a very strong distinction between the three types of epistemic emotions.

**Table 3:** The variance components of each factor and their interactions

Participant	34.6%
Video	6.2%
Emotion type	-
Participant x video	39.0%
Video x emotion type	0.2%
Participant x emotion type	4.9%
Participant x video x emotion type	15.0%

We also examined the extent to which these ratings can be explained by the individual difference variables we assessed (i.e., age, gender, general interest in magic tricks, and experience in performing the trick) by including these variable as additional fixed-effect predictors in the model. We also included a fixed-effect of trial number and its random slopes as an additional within-person predictor to explore the potential role of familiarization in epistemic emotions. Table 4 reports the results. Across epistemic emotions there is a consistent age effect, suggesting that older participants tended to have higher overall epistemic emotions, (βs = 0.02, *p*s < .01). On the other hand, confidence was negatively associated with age (β = – 0.02, *p* < .01). There were no statistically significant gender differences (– 0.20 ≤ βs ≤ 0.03, *p*s >.05). General interest in magic tricks is significantly and positively associated with epistemic emotions and confidence (0.16 ≤ βs ≤ 0.35, *p*s < .001). Experience with magic tricks has a strong positive association with confidence (β = 1.17, *p* < .001), and a weak positive relationship with interest (β = 0.38, *p* < .05). Finally, number of trials was negatively associated with epistemic emotions and confidence, indicating that there was a general declining trend for these ratings over trials (– 0.14 ≤ βs ≤ (– 0.08, *p*s < .001).

**Table 4:** Fixed effects (standard errors) predicting ratings of epistemic emotions in mixed-effects modeling

	Surprise in response to the trick	Interest in the trick	Confidence in the solution	Curiosity in the solution
Intercept	3.29 *** (0.36)	3.35 *** (0.33)	4.17 *** (0.37)	3.02 *** (0.38)
Trial number	– 0.08 *** (0.02)	– 0.12 *** (0.02)	– 0.11 *** (0.02)	– 0.14 *** (0.02)
Age	0.02 ** (0.01)	0.02 ** (0.01)	– 0.02 ** (0.01)	0.02 ** (0.01)
Gender (male:0, female:1)	0.03 (0.16)	– 0.07 (0.15)	– 0.20 (0.17)	– 0.03 (0.17)
General interest in magic	0.26 *** (0.04)	0.31 *** (0.04)	0.16 *** (0.04)	0.35 *** (0.04)
Experience in performing magic	0.26 (0.19)	0.38 * (0.18)	1.17 *** (0.20)	0.19 (0.21)

*p < .05, ** *p* < .01, *** *p* < .001

To further examine the differentiation of these epistemic emotions, we also computed the correlations between surprise in response to the trick, interest in the trick, confidence in the solution, and curiosity in the solution at the within-person level. More specifically, we calculated within-person correlations for each participant (using video clips as the unit of analysis) and then computed the mean and SD of the correlations across participants (please see Table 5). Surprise in response to the trick, interest in the trick, and curiosity in the solution were highly correlated; however, there are also considerable individual differences (i.e., SD is relatively high), indicating that these three epistemic emotions are overlapping but distinct concepts, especially for particular individuals (Fayn et al. 2019). The distributions of the within-person correlations were all unimodal but the distribution was also substantially skewed given the limit of correlation coefficients (– 1 ≤ r ≤ 1) and large individual differences. For completeness, we also computed a between-person correlation and have reported it in Appendix Table 8. As is typical with correlations of aggregated scores (Robinson, 1950), the between-person correlation between epistemic emotions is very high.

**Table 5:** Means of within-person correlations among ratings

	1	2	3
1: surprise in response to the trick			
2: interest in the trick	0.75 (0.27, – 2.43)		
3: confidence in the solution	– 0.45 (0.36, 1.16)	– 0.38 (0.36, 1.11)	
4: curiosity in the solution	0.67 (0.27, – 1.99)	0.70 (0.26, – 2.00)	– 0.42 (0.39, 1.09)

Note: All correlations are statistically significant (*p*s<.001). *Numbers in parentheses* represent SD and skewness across participants.

### Stimulus availability

The final set of MagicCATs video clips is available upon request for research purpose. The request procedure is posted to Open Science Framework (https://osf.io/ad6uc/) along with the stimulus list (equivalent to Tables 6 and 7) and raw rating data.

## Discussion

The current article introduces 166 magic trick video clips (MagicCATs) as a novel stimulus set to induce epistemic emotions. MagicCATs include a variety of magic tricks with various lengths (8–155 s) and diverse materials (e.g., playing cards, coins, sponges etc.), making it easy for researchers to select and use the subset of stimuli best suited to their own research purposes. Furthermore, rating results showed sufficient within-person variance with moderate mean levels of epistemic emotions, meaning that these video clips are suitable to examine these emotions on a trial-by-trial basis. MagicCATs video clips are available for research purpose, and stimulus list and raw rating data are also available online (please go to https://osf.io/ad6uc/).

The mixed-effects modeling analysis indicated that there is substantial within-person variation of the ratings of epistemic emotions across different video clips. This is a useful property for an experimental stimulus set and these results demonstrated its capability to capture within-person variation of these emotions. However, the majority of this within-person variance came from participant x video clip effects, meaning that there are substantial individual differences in which magic tricks were found surprising, curious, and interesting by participants (see Fastrich et al., 2017 for a similar finding with trivia questions). Therefore, when examining epistemic emotions with these stimuli it may be ideal to assess these epistemic emotions on a participant-by-participant basis. Of course, video variance was still present, and therefore, it is possible for future studies to rely on the aggregated ratings reported in the Appendix Table 7 to compare, for example, response between high vs. low curiosity magic video clips. However, the results should be interpreted with caution given the potential for large individual differences. It is worth noting that we also observed large participant variance that is comparable to the participant x video variance (Table 3). This variance may reflect general response bias but suggest possible individual differences in the overall tendency for participants to experience epistemic emotions.

One important research question for the future is to identify the source of these large individual differences. As the first step, we explored some basic demographic variables that we assessed in this experiment. First, older participants tended to have higher overall epistemic emotions. These findings are consistent with previous studies using trivia questions (Fastrich et al., 2017; McGillivray et al., 2015) but somewhat contradictory with a declining trend in curiosity-related personality traits in older age groups (Sakaki, Yagi, & Murayama, 2018). On the other hand, confidence was negatively associated with age, which is also consistent with the previous study (Jay, 2016). Second, there were no statistically significant gender differences. Gygax et al. (2019) reported that males are more motivated to discover how tricks are done than females, which is inconsistent with our results for curiosity and confidence in solution. There are some methodological differences (e.g., the participants were asked to report the solution they had guessed in their study) and the future research would be necessary for clarity in what gender differences exists in which contexts. It is possible that other basic demographic variables such as culture can explain individual differences. Unfortunately, we only collected the data from US participants and could not analyze potential cultural differences. Because we provide all the experimental materials and programs online (code for the experiment is also available on request), we are hoping that those who are interested in the topic will conduct follow-up studies with different populations to examine the role of culture in the subjective experiences of epistemic emotions. Note that, using generalizability theory, our variance decomposition estimates (Table 2) allow researchers to determine the number of video clips to be used to reliably assess such individual differences (see Brennan, 2001 for formulas).

It is worth noting that the curiosity and interest ratings used in the current experiment may have slightly different meanings than those used in other studies on curiosity and interest. Specifically, curiosity ratings in the current experiment asked whether participants were curious about how the trick was done (i.e., curiosity in the solution), whereas interest ratings asked whether they were interested in the magic tricks themselves (i.e., interest in the trick). Curiosity in the solution focused on the subjective motivation to close the knowledge gap (Loewenstein, 1994), while interest in the trick focused more on the positive emotional feelings due to the apparent uncertainty and impossibility of the trick (Silvia, 2005). However, some other studies (e.g., Fastrich et al., 2017; McGillivray et al., 2015) operationalized the feeling of interest as the satisfying of curiosity (e.g., the positive feelings when seeing the answer of a trivia questions). Some other researchers, especially in the field of education, define interest more broadly in relation to learner's goals, values, and pre-existing knowledge (for a review, see Hidi & Renninger, 2019). Our labeling of curiosity and interest are rather ad hoc: we simply used the terms in a way that participants can intuitively understand the focus of these feelings, i.e., curiosity in the solution vs. interest in the magic trick. In fact, we are hesitant to commit to the debate over the exact definitions of curiosity and interest (for details on our view, see Murayama, Fitzgibbon, & Sakaki, 2019). However, researchers should bear in mind differences in the conceptualizations of curiosity and interest when interpreting the findings reported in the current article (see also Shin & Kim, 2019).

Some additional points are worth discussing. First, our findings from the rating analysis may have a limitation in that we relied on a single-item measure to assess epistemic emotions, which are expected to be less reliable than multiple-item measures. However, we believe that simple subjective emotional feelings such as surprise can be assessed reliably and validly with a single-item measure (see also Diamantopoulos et al., 2012), and in fact most of the previous studies used a single-item measure to assess such epistemic emotions and found meaningful relations with other variables (e.g., Kang et al., 2009; Vogl et al., 2019). Even so, we need to wait for an empirical investigation to understand the extent of the problem when using single-item measures to examine epistemic emotions. Second, although we focused on the emotions of curiosity, interest, and surprise, other types of emotions may be experienced when people see magic tricks. For example, Leddington (2016) argued that the heart of the experience of magic is a conflict between “intellectual belief (the magic is impossible)” and “emotional belief (the magic is actually happening).” Further research would be required to investigate such emotions as well. Third, we observed that the number of trials was negatively associated with epistemic emotions and confidence, indicating that there was a general declining trend for these ratings over trials, perhaps reflecting a familiarization effect. Researchers who use MagicCATs should take care of this declining trend when they decide how many tricks they use in their studies. Fourth, the variance decomposition analysis and within-person correlation suggest that the three types of epistemic emotions we assessed are substantially overlapping even if they also exhibited some unique variances. The high inter-correlation between epistemic emotions is consistent with previous studies and not surprising given that these epistemic emotions are likely to be the causes or consequences of one another (e.g., Vogl et al., 2019, 2019). As all the ratings were assessed soon after each other, response bias might have played some role too (Podsakoff et al., 2003). However, these findings also suggest the importance of assessing and including these emotions together in an empirical study when researchers are interested in examining unique aspects of each specific type of epistemic emotions. Finally, although we did our best to control for various aspects of the video clips (e.g., background, expression of magicians, etc.), there are notable differences between the magic tricks. For example, running times vary widely between the video clips, and there are some video clips which have subtitles and/or show a third person (e.g., a person to pick a card). These factors can be confounding in experimental work. However, these variations were necessary in order to ensure the generalizability of experimental findings from these stimuli. Researchers can easily control the differences between the videos by pre-screening the video clips according to the aims of studies. We are hoping to expand the collection further so that it is easier for researchers to select video clips that fit well with their research questions.

## Appendix

**Table 6:** Detailed information about MagicCATs

Magic ID	Name	Credit	Phenomena Category	Materials	Length	Subtitle	Brief description
K1	Deck vanish	Unknown	Disappearance	Cards	0:00:18	No	The deck vanishes into a black box
K2	Color changing book	Unknown	Color change	Book	0:00:26	No	The sweets from a page in a children's story book are poured out onto the table from it
K3	Ladybag magic (Paddle move)	Unknown	Color change	Knife	0:00:25	No	A magician has a leaf on which two ladybirds appear on either side before magically disappearing
K4	Insta Cube	Craig Nichols	Other	Rubix Cube	0:00:30	No	The magician magically solves the Rubix Cube by spinning it in the air
K5	The Last Trick of Dr. Daley_ver1	Jacob Daley	Exchange	Cards	0:00:35	Yes	Magician has four aces and places the two red cards on the table but when he reveals the cards, he is actually holding the red cards and the black cards are on the table
K6	Ace assembly	Alex Elmsley	Transportation	Cards	0:00:41	Yes	An ace is placed onto the table and three cards on top - all of which turn out to be aces
K7	Card Warp	Roy Walton	Other	Cards	0:00:33	No	Magician has a black and a red card. He folds the red card in half and then does the same with the black card before slotting the black card in between the folding of the red one. He folds the cards back on themselves and then pulls the card in the middle out revealing that the middle card is now the red one. He folds them back once again, revealing that it is in fact the black card in the middle
K8	Card change	Unknown	Color change	Cards	0:00:08	No	Card changes with a small gesture
K9	Four aces location	Unknown	Assembly	Cards	0:00:50	No	Magician deals four decks onto the table each starting with an ace. It turns out all the aces end up in one pile and the others are made up of the same cards
K10	linking pin	Dan Garrett	Penetration	Safety pin	0:00:31	No	Safety pins magically interlock without breaking the seal
K11	Stargazer	Alan Wong	Change	Rubber band	0:00:25	No	Magician has two elastic bands. He makes several star shapes using them and then produces a star shaped elastic band
K12	Card toon	Dan Harlan	Take one	Cards	0:00:33	No	Volunteer chooses 7 of hearts. The magician then shows the audience the flip book story on the cards which shows that a fairy also chooses the 7 of hearts
K13	Crazy Man's Handcuffs	Michael Ammar	Penetration	Rubber band	0:00:19	No	Magician has two elastic bands and tangles them up. They then become untwisted
K14	Quick as a wink	Unknown	Take one	Cards	0:00:35	Yes	Volunteer selects 2 of diamonds and places it back in the deck. Then he sandwiches the deck with a Joker on each end. Then he throws the card from one hand to the other and sandwiched between the two Jokers is the volunteer's selected card
K15_Long	Chicago Opener	Al Leech and Frank Everhart	Take one/ color change	Cards	0:01:13	Yes	Volunteer selects a card and put it back to a deck then this card becomes a colored card
K15_Short	Chicago Opener	Al Leech and Frank Everhart	Take one/ color change	Cards	0:00:37	No	Volunteer selects a card and put it back to a deck then this card becomes a colored card
K16_Long	Ambitious Card	Gustav Alberti	Transportation	Cards	0:01:12	Yes	Volunteer selects and signs a card. Magician put it inside the deck and the signed card appeared on the top repeatedly
K16_Short	Ambitious Card	Gustav Alberti	Transportation	Cards	0:00:31	No	Volunteer selects and signs a card. Magician put it inside the deck and the signed card appeared on the top repeatedly
K17	Slop Shuffle	Sid Lorraine	Take one	Cards	0:00:46	No	Volunteer selects a card and the magician shuffled a deck. This card is the only one that has the opposite side
K18	3D advertising	Henry Evans	Materialization	Cards	0:00:22	No	Pack of cards comes out of a piece of paper
K19_Long	Chop Cup	Al Wheatley	Transportation	Cups and balls	0:00:31	No	balls in a cup warps and turns into a toy rabbit
K19_Short	Chop Cup	Al Wheatley	Transportation	Cups and balls	0:00:19	No	balls in a cup warps and turns into a toy rabbit
K21_Long	Bizarre Twist_ver1	Paul Harris	Color change	Cards	0:00:43	No	A card flips with a shorthand gesture
K21_Short	Bizarre Twist_ver1	Paul Harris	Color change	Cards	0:00:17	No	A card flips with a shorthand gesture
K22_Long	Three-card Monte	Shoji Takahito	Exchange	Cards	0:00:43	No	Volunteer is shown three cards; one red and two black cards. The magician places the cards face down on to the table in the same order that he presented the cards to the volunteer. The magician then asks the volunteer to point to the red card but when the magician turns the card over it is in fact a black card
K22_Short	Three-card Monte	Shoji Takahito	Exchange	Cards	0:00:23	No	Volunteer is shown three cards; one red and two black cards. The magician places the cards face down on to the table in the same order that he presented the cards to the volunteer. The magician then asks the volunteer to point to the red card but when the magician turns the card over it is in fact a black card
K23	Wonder Pen A Tration	Doug Edwards	Penetration	Pen/ Bill	0:00:35	No	Magician puts a hole through the money bill but then reveals that the hole disappears
K24	Bill switch	Unknown	Color change	Bill	0:00:24	No	£10 note is transformed into a £20
K25	Spellbound	Dai Vernon	Color change	Coin	0:00:15	No	Coin is changed back and forth from silver to copper
K27	Rising card	Unknown	Psychokinesis	Cards	0:00:26	No	Volunteer is asked to choose a card from the deck. They choose a card and then place it back into the middle of the deck. The magician then shuffles the card and waves his hand over the deck as the chosen cards rises from the pack
K28	Bottle from paper bag	Unknown	Appearance	Bottle	0:00:13	No	Magician produces a water bottle from a paper bag
K29	Bottle production 1	Unknown	Appearance	Bottle	0:00:15	No	Magician produces a water bottle from a silk
K30	Bottle production 2	Unknown	Appearance	Bottle	0:00:13	No	Magician produces a water bottle from his jacket
K31	HANASAKI-crystal	Sugawara Shigeru	Appearance	Flower	0:00:12	No	Magician produces a flower from a glass box
K32	Billiard Ball 1	Unknown	Appearance	Billiard Ball	0:00:11	No	Magician produces a red ball from his hand
K33	Billiard Ball 2	Unknown	Color change	Billiard Ball	0:00:11	No	Magician turns a red ball into a white one using hand gestures. Then he turns it back into the red one
K34	Rope through body	Unknown	Penetration	Rope	0:00:11	No	Magician ties rope around neck but manages to untie it
K35_Long	Professor's nightmare	Bob Carver and Hen Fetsch	Other	Rope	0:00:27	No	Magician has three ropes of various different lengths and manages to make them all the same size
K35_Short	Professor's nightmare	Bob Carver and Hen Fetsch	Other	Rope	0:00:13	Yes	Magician has three ropes of various different lengths and manages to make them all the same size
S1	Play it straight	John Bannon	Take one	Cards	0:00:52	No	Magician shuffles card, revealing one suit is facing upwards throughout the deck and is in order
S2	Quick Coincidence	Unknown	Take one	Cards	0:00:32	No	Volunteer selects four cards, all of which turn out to be the aces
S3_Long	Bizarre Twist_ver2	Paul Harris	Color change	Cards	0:00:38	No	Card flips or changes to another color with a quick gesture
S3_Short	Bizarre Twist_ver2	Paul Harris	Color change	Cards	0:00:20	No	Card flips or changes to another color with a quick gesture
S4	Torn & Restored Transpo	David Williamson	Restoration/Take one	Cards	0:00:46	Yes	Magician shows the audience a card which he then rips up. Then he shows the audience another card, but this time waves the card over the ripped up one, revealing that the two cards have swapped places
S5	Drible force	Unknown	Mind reading	Cards	0:00:22	No	The magician verbally points to the card that was seen by volunteer
S6	1 to 4	Unknown	Appearance	Cards	0:00:21	No	Volunteer selects one card which then magically turns into four - all of which are the same e.g., all Queens
S7	Four cards change	Unknown	Change	Cards	0:00:13	Yes	Four different cards change to four kings
S8	Princess card trick	Henry Hardin	Mind reading	Cards	0:00:34	Yes	Six cards are placed on the table and the audience is asked to remember one of the cards. The magician then removes all of the cards from the table, laying one of the cards to the side but face down. He then places the cards back down on the table again revealing that the audience member's chosen card is no longer in the deck
S9	Blanc Deck	Unknown	Color change	Cards	0:00:32	No	Magician turns a deck of cards into a blank deck
S10	Ultra Visual Nightshades	Mark Allen and Paul Harris	Transportation	Bill	0:00:33	No	Magician draws glasses onto Queen on £10 note and then makes the glasses move
S11	Cigarette through the coin	Unknown	Penetration	Cigarette/ Coin	0:00:24	No	Magician pushes a cigarette through a coin and then reveals that there is no hole
S12	Moving a hole	L. Vosburgh Lyons	Transportation	Cards	0:00:41	No	A hole is hole-punched into the corner of a card. The magician then moves the hole to a different corner and then reveals that it's actually a black spot on the card
S13	Coins across	Unknown	Transportation	Coin	0:00:14	No	Magician has a coin in each of his hands. They both end up in one hand
S14	Powder sugar to block sugar	Unknown	Change	Sugar	0:00:26	No	Magician turns powdered sugar into a block of sugar
S15	Ring through the glass	Unknown	Penetration	Ring/ Grass	0:00:31	No	Magician places ring on the stem of a glass without breaking it
S16	Gipsy thread	Unknown	Restoration	Thread	0:00:45	No	Magician cuts piece of string in several places and then magically puts it back together whole.
S17	Multum in parvo	Bazar de Magia	Other	Milk/ Grass	0:01:14	No	Magician pours milk from a small glass into a bigger one but still manages to fill the bigger glass full of milk. He continues this process with bigger glasses each time and still manages to fill the bigger glasses.
S18	Boxes from Paperbag	Unknown	Appearance	Box	0:00:31	Yes	Magician pulls out 3 boxes with flowers in from a paper bag
S19	flow	Dan Hauss	Other	Bottle	0:00:44	Yes	Magician asks volunteer to hold hand over a water bottle. When turned upside down the water does not pour out until the magician gestures
S20	Giant Appearing Straw	Unknown	Appearance	Straw	0:00:18	No	Two magicians pull out giant straw from a brown paper bag
S21	Spoon bending	Unknown	Bending	Spoon	0:00:21	No	Magician bends a spoon using fingers
S22	Bill in lemon	Emil Jarrow and Doc Eason	Transportation	Bill/Lemon	0:01:10	No	Magician asks volunteer to sign piece of paper which then magically appears inside a lemon
S23	Thunderbird	Lee Asher	Appearance	Cards	0:00:16	Yes	Cards magical appear in magicians’ hand
S25	Handkerchief in Bread	Unknown	Transportation	Handkerchief/Bread	0:00:52	No	Handkerchief appears from bread
S26	Rope act	Unknown	Restoration	Rope	0:00:24	No	Magician cuts rope with fingers and then mends the broken rope
S27	Water Vanish	Unknown	Disappearance	Water	0:00:31	No	Magician pours water into cup from bottle and then pours the water into another cup but when he tips it upside down, no water spills out
S28	Dresscode	Calen Morelli	Change	T-shirts	0:00:16	No	Magician changes t-shirts very quickly when he turns around
S29	sticky situation	Andy Leviss	Restoration	Chewing gum	0:00:25	No	Magician transforms a chewed piece of gum back into its packaging
S30	Chinese Stick	Unknown	Other	Sticks	0:00:25	No	Magician pulls strings through a stick which makes the other string in the other stick move
S31	Wari-vanish	Takahito Shoji	Disappearance	Chopsticks	0:00:19	No	Magician places a pair of chopsticks into envelope and proceeds to crush the envelope and its contents into a paper ball
S32	Self-Tying Shoelace	Jay Noblezada	Other	Shoes	0:00:14	No	Magician ties his shoelace without using his hands
S33_Long	Swallowing balloon	Steve Bedwell	Other	Balloon	0:00:52	No	Magician eats a long balloon while it is full of air
S33_Short	Swallowing balloon	Steve Bedwell	Other	Balloon	0:00:45	No	Magician eats a long balloon while it is full of air
H1	Reduction	Nicholas Lawrence	Disappearance	Cards	0:00:12	No	A deck of cards reduced to 10
H2	The brade	Katsuya Masuda	Restoration	Cutter knife/Bill	0:00:46	No	Magician folds a £10 note in a piece of paper and cuts through them with a knife. He reveals that the £10 is still intact afterwards
H3	Coin assembly	Unknown	Assembly	Coins/ Cards	0:00:24	No	Magician hides four coins under four cards and reveals that the coins swap places under the cards in several different ways
H4_Long	Coin assembly & Reverse	Unknown	Assembly	Coin/Cards	0:00:57	No	Magician has four coins and two cards. He places the coins in each corner of the table and places two cards on top of the two top coins. He picks up one of the two remaining coins and makes it disappear from his hand and then reveals it is now underneath one of the cards with the other coin
H4_Short	Coin assembly & Reverse	Unknown	Assembly	Coin/Cards	0:00:21	No	Magician has four coins and two cards. He places the coins in each corner of the table and places two cards on top of the two top coins. He picks up one of the two remaining coins and makes it disappear from his hand and then reveals it is now underneath one of the cards with the other coin
H5	The ten count	Unknown	Transportation	Sponge	0:00:12	No	Two sponge balls each behind one hand end up in one hand
H6	Ball to Cube sponge	Unknown	Change	Sponge	0:00:15	No	Two sponge balls turn into a cube
H7	Salt and Silver	Giovanni Livera	Transportation	Coin	0:00:33	No	Coin appears everywhere around the salt case
H8	Capture	Luke Dancy	Other	Coin/ Smartphone	0:00:40	No	Magician puts coin into phone and then the coin comes out of the phone and into the volunteers’ hand
H9	Touch	Hanson Chien	Transportation	Rubber band	0:00:18	No	The magician has an elastic band on his hand which then magically ends up on the volunteers’ hand
H10	God hand	Lubor Fiedler	Restoration	Cards	0:00:20	No	Card that's split into four pieces is magically put back together
H11	V-Rank Card	Katsuya Masuda	Other	Cards	0:00:25	Yes	Magician shows a card with a red back and a blank back. Then he changes the blank back into the red back and vice versa
H12	One hand color change	Shin Lim	Color Change	Cards	0:00:08	No	The magician waves his hand over the card placed on the table which changes to a different card
H13	Frozen	Adam Grace	Other	Coin	0:01:09	No	Magician takes a coin from volunteer and freezes it in a paper napkin
H14	Insane	Andy Newman	Prediction	Cards	0:01:11	No	Magician has the King of hearts as a prediction and asks the volunteer to shuffle the deck. He then asks her to deal cards and stop when she likes. The card she stopped is the King of hearts
H15	Unbalance	Creis Co. ltd	Other	Toothpick	0:00:31	Yes	Magician is able to balance a toothpick on a deck of cards but the volunteer cannot
H16	Healed and sealed	Anders Moden	Restoration	Can	0:00:33	No	Magician pours coke into glass from an empty can
H17	Guillotine	Unknown	Other	Guillotine	0:00:46	Yes	The magician uses a guillotine to slice breadsticks, then asks the volunteer to put her finger in. He slices the breadstick but not her finger
H18	Fish appearance	Unknown	Appearance	Fish/ Bill	0:00:24	No	The magician unfolds a £10 note and drops a fish into a glass of water
H19	Sponge appearance	Tani Hideki	Appearance	Sponge	0:00:20	No	Magician places ball of paper in participants hand and sets it alight, revealing a red sponge ball
H20	Sponge routine	Unknown	Transportation	Sponge	0:00:20	No	Magician places on ball in the volunteer's hand and one in his own hand but reveals that both end up in the volunteer's hand
H21	Mind power deck	John Kennedy	Mind reading	Cards	0:01:01	No	Volunteers each remember one specific card from the deck shown to them by the Magician. The magician then reads (out loud) the names of the cards that the volunteers remembered
H22	Sixth sense	Henry Evans	Mind reading	Cards	0:01:26	No	This one is labelled as take 1
H24	Self-folding bill	Stefan Schutzer	Psychokinesis	Bill	0:00:13	No	A £10 note folds itself into half
H25	X change	Julio Montoro	Materialization	Cards/Coin	0:00:59	No	Volunteer signs card and magician then draws a coin on the back. The picture of the coin then turns into a real-life coin
H26	Tagged	Richard Sanders	Penetration	Cards/ Necklace	0:00:39	No	Volunteer selects a card which then sticks to the magician’s necklace at the end
H27	UFO card	Unknown	Floating	Cards	0:00:08	No	Card flies around magician in a circle
H28	Cardcase color change	Shin Lim	Color change	Card case	0:00:15	No	Card case changes color
H29	4 by 4	Shin Lim	Color change	Cards	0:00:20	No	four red cards change into four blue ones
H31	Big bang	Krisjian Pipho	Psychokinesis	Light Bulb	0:00:16	No	Magician breaks light bulb in a bag
H32	Tanging	Unknown	Disappearance	Cigarette	0:00:22	No	Magician makes a cigarette disappear and then reappear
H33	Envy-lope	Brandon David and Chris Turchi	Disappearance	Cards	0:00:42	No	Volunteer signs a card which the magician then ends up pulling out of an envelope
H34	iVanish	Ben Seidman	Disappearance	Coin	0:00:25	No	Magician places coin into eye and then pulls it out of the other one
H35	Indecent	Wayne Houchin	Penetration	Cards/ Ziplock	0:00:36	No	Volunteer signs card which the magician ends up pulling out of a Ziploc bag
H36	Macro psychic	Creis Co. ltd	Psychokinesis	Bolt	0:00:28	Yes	Screw and bolt unscrew itself
H37	Finger drop revolution	PROMA Web Shop	Other	Finger	0:00:28	Yes	Magician unscrews his finger and then shakes it back into place
H38	Floating cigarette	Steve Fearson	Floating	Cigarette	0:00:24	No	A cigarette floats around the magician
H39	Passin' Thru	Kevin Parker	Penetration	Coin/ Bottle	0:00:31	No	Magician puts coin into a glass bottle without it breaking
H40	Levitator	Andrew Mayne	Floating	Body	0:00:14	Yes	Magician levitates from on top of a box to the ground
H41	Sandwich Vanish	Shin Lim	Disappearance	Cards	0:00:16	No	Card on the table is magically changed into a different card with a quick gesture
Trick1	How to cheat at poker	Alvaro Argente	Color change	Cards	0:00:30	No	The magician shuffles the cards and turns four random cards into 4 kings
Trick2	Finding aces with mistake	Alvaro Argente	Assembly	Cards	0:00:53	No	The magician shuffles the cards and finds the four aces in the pack
Trick3	Triumph	Dai Vernon	Take one	Cards	0:00:50	Yes	A card trick where some of the cards end up face down and the other half face up and the chosen card is found in the middle
Trick4	Pressure	Daniel Garcia	Penetration	Balloon/ Smart phone	0:00:42	No	The magician puts a mobile phone inside a balloon with a simple gesture of his hands
Trick5	Smoke in the veins	Alan Rorrison	Other	Smoke	0:00:25	No	The magician draws a dot on the palm of his hand and then uses a lighter to make a gesture under his hand. The smoke then travels up his arm and out through his mouth
Trick6_Short	Chop cup	Don Alan	Transportation	Cup and ball	0:00:27	No	The classic cup and ball routine using only 1 cup and an egg
Trick6_Long	Chop cup	Don Alan	Transportation	Cup and ball	0:01:04	No	The classic cup and ball routine using only 1 cup and an egg
Trick7_Short	Multiplying sponge balls	Lybarger	Transportation	Sponge	0:00:20	No	The magician gets the volunteer to hold a foam ball which then doubles in to two. He repeats the trick and they then turn into three
Trick7_Long	Multiplying sponge balls	Lybarger	Transportation	Sponge	0:00:34	No	The magician gets the volunteer to hold a foam ball which then doubles in to two. He repeats the trick and they then turn into three.
Trick8_Short	Icebreaker	Alvaro Argente	Take one	Cards	0:00:39	Yes	The volunteer chooses a card and places it into the deck. The magician then shuffles the card, revealing to the camera that the selected card is not on the top nor the bottom. Then with a gesture of his hand, he transforms the top card into the selected card.
Trick8_Long	Icebreaker	Alvaro Argente	Take one	Cards	0:00:56	Yes	The volunteer chooses a card and places it into the deck. The magician then shuffles the card, revealing to the camera that the selected card is not on the top nor the bottom. Then with a gesture of his hand, he transforms the top card into the selected card.
Trick9	The Last Trick of Dr. Daley	Jacob Daley	Exchange	Cards	0:00:26	No	The magician places two red cards down and then reveals that they are actually two black cards
Trick10	Aces color change	Edward Marlo and Alvaro Argente	Color change	Cards	0:00:52	No	The magician has four aces with red backs. He then turns the back color to blue. Eventually he reveals that all of the aces have a different color back
Trick11	Poker face	W. Ciuro	Take one	Cards	0:01:18	No	The volunteer selects a card and places it back into the deck. The magician shuffles the cards and then places four cards on the table. One of the cards is the selected card. The magician then places a further 4 cards on the table and reveals that the 4th card is actually the selected card. Then he reveals that the other cards are not the selected card
Trick12_Short	Sandwich effect	Harry Lorayne	Transportation	Cards	0:00:34	No	The magician reveals that the selected card is sandwiched between two cards
Trick12_Long	Sandwich effect	Harry Lorayne	Transportation	Cards	0:01:46	No	The magician reveals that the selected card is sandwiched between two cards
Trick13	Inside a box	Dani Daortiz	Take one	Cards	0:00:44	No	The volunteer examines an empty card box and then chooses a card from the magicians’ deck. With a gesture, the magician reveals that the card was in the box
Trick14_Short	Rub-a-dub Vanish	Paul Stadleman	Take one	Cards	0:00:37	No	The volunteer chooses a card and places it back into the deck. With a gesture, the magician reveals the selected card
Trick14_Long	Rub-a-dub Vanish	Paul Stadleman	Take one/Disappearance	Cards	0:00:47	No	The volunteer chooses a card and places it back into the deck. With a gesture, the magician reveals the selected card. The magician then makes the selected card disappear
Trick15	Invisible deck	Jor Berg	Prediction	Cards	0:00:37	No	Card a volunteer picked up. In another deck, the card appeared face down while the other cards are face up
Trick16_Short	Signed Transpo	Alvaro Argente	Take one	Cards	0:00:59	No	Four cards (two red and two black) are placed on top of the table. A participant signs a card and places it back into the pile. Then with a gesture, the magician flicks the signed card into the four cards on the table
Trick16_Long	Signed Transpo	Alvaro Argente	Take one	Cards	0:01:42	No	Four cards (two red and two black) are placed on top of the table. A participant signs a card and places it back into the pile. Then with a gesture, the magician flicks the signed card into the four cards on the table. The magician then loses the car and finds it again in his pocket
Trick17	MST	Mathew Dowden	Other	Cards	0:01:55	No	Volunteer signs the front of one card and the back of another. The magician transfers the signatures onto the front and back of one card
Trick18_Long	Ambitious card	Gustav Alberti	Transportation	Cards	0:01:22	No	The magician signs a card and returns it to the middle of the deck. He clicks his fingers and reveals the card is now on top. Then he bends the card and places it back into the middle. After a gesture, the card ends up on top again
Trick18_Short	Ambitious card	Gustav Alberti	Transportation	Cards	0:00:31	Yes	The magician signs a card and returns it to the middle of the deck. He clicks his fingers and reveals the card is now on top. Then he bends the card and places it back into the middle. After a gesture, the card ends up on top again
Trick19	Card in the mouth	Unknown	Transportation	Cards	0:01:03	No	The volunteers selected card appears in the magician's mouth
Trick20_Short	Nut and rope	Giovanni Livera	Penetration	Nut/ Rope	0:00:36	No	The magician shows a metal nut and rope. It doesn’t matter how many times he secures the nut with the rope, it always scape
Trick20_Long	Nut and rope	Giovanni Livera	Penetration	Nut/ Rope	0:01:17	No	The magician shows a metal nut and rope. It doesn’t matter how many times he secures the nut with the rope, it always scape
Trick21	Magic ring	Wayne Dobson	Penetration	Ring/ Key	0:01:09	No	The magician links the volunteers ring onto a key and then unlinks it
Trick22_Long	Cups&Balls	David Forrest	Penetration	Cups and balls	0:01:06	No	Three shot glasses are placed on the table and small pieces of paper scrunched into balls are put on top. The magician then shuffles the shot glasses around revealing the fire paper underneath has multiplied. Eventually he reveals a red ball underneath one of the shot glasses
Trick22_Short	Cups&Balls	David Forrest	Penetration	Cups and balls	0:00:33	Yes	Three shot glasses are placed on the table and small pieces of paper scrunched into balls are put on top. The magician then shuffles the shot glasses around revealing the fire paper underneath has multiplied. Eventually he reveals a red ball underneath one of the shot glasses
Trick23_Short	Ace to King	Pepe Carroll and Alvaro Argente	Other	Cards	0:00:28	No	Four aces and two jacks are removed from the deck. One ace is placed on the table and the magician then finds the next cards and places them in order starting from the ace
Trick23_Long	Ace to King	Pepe Carroll and Alvaro Argente	Other	Cards	0:02:35	No	Four aces and two jacks are removed from the deck. One ace is placed on the table and the magician then finds the next cards and places them in order starting from the ace
Trick24	Cut by the card	Unknown	Take one	Cards	0:00:29	No	Find a card that the volunteer picked up
Trick25	Do as I do	Vicente Canuto	Take one	Cards	0:01:12	No	The magician gets the volunteer to copy his card shuffling and cutting. The volunteer ends up finding their card in the opposite deck
Trick26	Copy cat	Unknown	Take one	Cards	0:00:53	No	The magician searches spectator's card by cutting deck
Trick27	Bell on a card	Alvaro Argente	Transportation	Cards	0:01:02	Yes	The volunteers selected card appears under the bell
Trick28	Chinese linking rings	Unknown	Penetration	Rings	0:00:44	No	Magician links and unlinks several silver rings
Trick29	Scotch and Soda	Richard Himber	Transportation/ Disappearance	Coins	0:01:05	No	The magician shows a silver and a copper coin. Every time he puts a coin in his pocket, the coin appears again in his hand. In the end, all the coins disappear from his hand
Trick30	Mental Bend	Unknown	Bending	Coin	0:00:56	No	The magician asks the audience for a coin. They signed it to be sure that is in fact their coin. Then the coins magically bend inside the closed fist of the audience
Trick31	Coin detection	Unknown	Take one	Cards/ Coin	0:00:38	No	A card is selected and lost in the deck. Then, the magician shows a coin and makes it disappear. When the cards are spread on the table, the coin can be seen in the middle of the extension… marking where the selected card is
Trick32_Long	One coin routine	David Roth	Transportation	Coin	0:00:42	No	The magician makes a silver coin disappear and reappear several times
Trick32_Short	One coin routine	David Roth	Transportation	Coin	0:00:12	No	The magician makes a silver coin disappear and reappear several times
Trick33	Pixel:	David Jade	Take one	Cards/ Rubber band	0:00:48	No	A card is selected and lost in the deck. Then, the magician found a selected card using rubber band
Trick34	Omni deck	Paul Harris	Change	Cards	0:00:55	No	Volunteer chooses and signs a card. The magician then places the deck in the volunteer's hand and finds the missing card. When the volunteer reveals the deck, it is in fact a clear block in the shape of a deck of cards
Trick35	Three card monte	Unknown	Transportation	Cards	0:00:20	No	The volunteer has to track a card and the magician keeps swapping it to the middle
Trick36_Long	Four Kings	Eddie Fetcher and Joshua Jay	Color change	Cards	0:01:00	No	Two kings change into two jokers. Then, the magician finds four kings in the deck and finally he reveals that the color of the deck is different
Trick36_Short	Four Kings	Eddie Fetcher	Color change	Cards	0:02:08	No	Two kings change into two jokers
Trick37	Impossible escape bands	Unknown	Transportation	Rubber bands	0:00:51	No	An elastic band visually travels from finger to finger, then it travels again even when the fingers are secured with another elastic band
Trick38	Classic Disappearance	Milbourne Christopher	Disappearance/ Appearance	Handkerchief	0:00:32	No	The magician closes his left fit around a silk, and makes it disappear. Then, he makes it appear from his right fist
Trick39	Tiny Plunger	Mathieu Bich and Jon Armstrong	Take One	Cards/ Tiny plunger	0:00:51	No	The magician uses small plunger to pick out the card
Trick40	Coin bite	Roy Kueppers	Restoration	Coin	0:00:23	No	The magician borrows a coin and visually “bites” a piece of it. Then, in a gesture, the coin is restored
Trick41	Silk Transformation	Russ Walsh	Change	Silk/ Cane	0:00:13	No	The magician shows a silk, and in a gesture, turns it into a metal cane

**Table 7:** Rating Means and SDs for individual magic tricks in MagicCATs

Magic ID	All data											Removing the trials that were unclear to participants.								
	Mean						SD					Mean					SD			
	N of participants	Clarity of the trick	Surprise in response to the trick	Interest in the trick	Confidence in the solution	Curiosity in the solution	Clarity of the trick	Surprise in response to the trick	Interest in the trick	Confidence in the solution	Curiosity in the solution	N of participants	Surprise in response to the trick	Interest in the trick	Confidence in the solution	Curiosity in the solution	Surprise in response to the trick	Interest in the trick	Confidence in the solution	Curiosity in the solution
K1	99	0.92	6.47	6.19	3.28	6.31	0.27	2.47	2.47	2.65	2.81	91	6.60	6.34	3.34	6.51	2.41	2.41	2.73	2.74
K2	101	0.92	6.19	6.50	4.21	6.42	0.27	2.72	2.71	2.99	2.88	93	6.39	6.61	4.30	6.47	2.62	2.63	3.01	2.79
K3	101	0.83	4.66	4.73	4.03	4.74	0.37	2.27	2.45	2.50	2.60	84	4.74	4.88	4.32	4.77	2.22	2.44	2.55	2.51
K4	101	0.90	6.28	6.49	3.42	6.56	0.30	2.49	2.46	2.61	2.59	91	6.45	6.74	3.37	6.70	2.46	2.40	2.67	2.55
K5	99	0.75	5.40	5.16	3.55	5.05	0.43	2.52	2.55	2.76	2.78	74	5.86	5.65	3.72	5.41	2.28	2.36	2.89	2.68
K6	102	0.73	4.94	5.06	3.16	5.08	0.45	2.54	2.68	2.47	2.75	74	5.36	5.64	3.55	5.46	2.34	2.44	2.51	2.58
K7	107	0.73	5.04	5.01	3.11	5.21	0.44	2.50	2.66	2.54	2.85	78	5.56	5.69	3.22	5.86	2.45	2.58	2.45	2.73
K8	99	0.47	3.74	3.56	3.17	3.73	0.50	2.54	2.48	2.62	2.71	47	4.62	4.66	4.55	4.64	2.65	2.64	2.75	2.94
K9	101	0.66	5.01	4.68	3.06	4.98	0.47	2.54	2.64	2.52	2.89	67	5.81	5.55	3.64	5.90	2.29	2.38	2.65	2.59
K10	99	0.90	5.11	5.36	4.13	5.28	0.30	2.62	2.60	2.82	2.83	89	5.31	5.53	4.18	5.60	2.63	2.54	2.77	2.77
K11	101	0.91	6.09	6.23	4.15	5.83	0.28	2.42	2.37	2.84	2.58	92	6.21	6.39	3.99	5.92	2.39	2.26	2.88	2.59
K12	99	0.86	6.68	6.78	4.31	6.37	0.35	2.87	2.87	3.16	3.07	85	7.20	7.20	4.14	6.82	2.61	2.66	3.17	2.87
K13	102	0.81	4.06	4.24	4.66	4.45	0.39	2.35	2.41	2.71	2.76	83	4.28	4.55	4.83	4.73	2.27	2.35	2.58	2.66
K14	107	0.87	5.51	5.59	3.36	6.05	0.34	2.47	2.45	2.61	2.61	93	5.67	5.85	3.47	6.23	2.48	2.39	2.64	2.55
K15_Long	99	0.87	6.28	6.32	3.09	5.97	0.34	2.79	2.72	2.43	2.91	86	6.65	6.74	2.87	6.33	2.69	2.59	2.41	2.86
K15_Short	95	0.91	5.98	5.99	3.56	6.42	0.29	2.70	2.69	2.77	2.77	86	6.08	6.19	3.63	6.64	2.73	2.66	2.80	2.71
K16_Long	95	0.85	5.89	5.98	3.11	6.05	0.35	2.67	2.75	2.43	3.04	81	6.15	6.23	3.05	6.36	2.60	2.64	2.44	2.94
K16_Short	99	0.82	4.36	4.44	3.67	4.61	0.39	2.49	2.52	2.79	2.68	81	4.53	4.65	3.77	4.77	2.56	2.60	2.77	2.76
K17	99	0.92	5.10	5.21	3.20	5.36	0.27	2.79	2.67	2.41	2.88	91	5.15	5.33	3.22	5.49	2.81	2.66	2.44	2.87
K18	101	0.76	5.18	5.00	4.10	5.33	0.43	2.62	2.74	2.89	2.86	77	5.57	5.51	4.32	5.81	2.50	2.49	2.94	2.70
K19_Long	99	0.90	6.57	6.26	3.96	5.93	0.30	2.64	2.68	2.87	2.96	89	6.46	6.18	4.06	5.91	2.67	2.62	2.87	2.97
K19_Short	95	0.88	5.36	5.33	4.56	5.64	0.32	2.45	2.29	2.88	2.60	84	5.44	5.43	4.74	5.85	2.46	2.26	2.95	2.54
K21_Long	95	0.85	6.38	6.17	3.54	6.24	0.35	2.56	2.56	2.72	2.84	81	6.58	6.28	3.41	6.43	2.56	2.53	2.63	2.78
K21_Short	99	0.61	4.26	3.92	3.65	4.25	0.49	2.49	2.42	2.87	2.39	60	4.67	4.62	4.23	4.80	2.48	2.37	2.91	2.48
K22_Long	99	0.94	4.92	5.54	4.28	5.63	0.24	2.78	2.62	2.99	2.84	93	4.82	5.49	4.20	5.60	2.79	2.62	2.99	2.85
K22_Short	95	0.88	5.13	5.16	4.88	5.56	0.32	3.10	2.95	3.07	3.13	84	5.15	5.19	5.05	5.61	3.09	2.91	3.10	3.12
K23	99	0.93	6.71	6.77	2.92	6.62	0.26	2.35	2.29	2.58	2.67	92	6.80	6.85	2.93	6.73	2.32	2.27	2.63	2.63
K24	101	0.83	4.75	4.98	4.37	4.96	0.37	2.54	2.50	2.85	2.72	84	5.17	5.36	4.65	5.26	2.41	2.34	2.79	2.57
K25	99	0.76	3.82	3.79	5.48	3.86	0.43	2.46	2.43	3.47	2.67	75	3.93	3.95	6.15	3.87	2.49	2.39	3.36	2.60
K27	102	0.93	4.76	5.06	4.15	5.38	0.25	2.62	2.59	2.73	2.72	95	4.80	5.18	4.15	5.44	2.62	2.58	2.78	2.72
K28	107	0.88	5.65	5.70	4.02	5.79	0.33	2.77	2.66	2.94	2.94	94	6.01	6.05	3.97	6.14	2.66	2.46	2.87	2.79
K29	99	0.93	5.96	5.68	4.18	5.54	0.26	2.73	2.77	2.91	3.02	92	5.85	5.54	4.28	5.41	2.75	2.74	2.91	3.03
K30	107	0.70	3.91	3.62	5.40	4.15	0.46	2.54	2.31	2.95	2.86	75	4.23	4.04	5.56	4.39	2.58	2.24	2.81	2.90
K31	99	0.93	5.22	5.22	4.95	5.31	0.26	2.91	2.82	3.20	2.94	92	5.32	5.30	4.95	5.48	2.87	2.80	3.16	2.91
K32	99	0.89	4.19	4.11	5.04	4.27	0.31	2.46	2.54	3.19	2.65	88	4.34	4.31	5.41	4.31	2.53	2.59	3.10	2.63
K33	99	0.91	4.84	5.14	4.70	5.08	0.29	2.45	2.59	2.97	2.73	90	4.96	5.33	4.79	5.30	2.44	2.52	3.01	2.72
K34	101	0.86	5.27	5.37	4.14	5.58	0.35	2.67	2.68	2.88	2.90	87	5.51	5.69	4.30	5.89	2.55	2.51	2.89	2.77
K35_Long	95	0.63	4.23	4.20	3.68	4.63	0.48	2.80	2.72	2.84	2.96	60	5.18	5.05	4.47	5.65	2.68	2.62	2.95	2.82
K35_Short	99	0.71	4.09	3.81	4.20	3.97	0.46	2.61	2.50	3.08	2.49	70	4.57	4.30	4.99	4.30	2.59	2.49	3.07	2.49
S1	107	0.83	6.08	5.82	2.98	6.03	0.37	2.86	2.88	2.43	3.01	89	6.57	6.39	3.08	6.44	2.64	2.65	2.47	2.88
S2	99	0.89	4.87	5.05	4.48	4.95	0.31	2.87	2.71	3.23	2.84	88	4.93	5.28	4.60	5.08	2.89	2.63	3.27	2.85
S3_Long	99	0.87	5.52	5.34	3.44	5.39	0.34	2.63	2.54	2.59	2.81	86	5.65	5.47	3.58	5.44	2.59	2.51	2.67	2.76
S3_Short	95	0.79	4.80	4.81	3.46	5.23	0.41	2.45	2.51	2.59	2.88	75	5.17	5.36	3.89	5.77	2.35	2.38	2.67	2.79
S4	101	0.84	7.21	7.38	2.61	7.41	0.37	2.46	2.50	2.16	2.68	85	7.65	7.88	2.62	7.89	2.02	1.95	2.22	2.20
S5	99	0.95	4.42	4.29	4.99	4.81	0.22	2.72	2.61	3.09	2.92	94	4.52	4.33	5.06	4.87	2.74	2.64	3.12	2.95
S6	101	0.89	4.98	5.20	4.33	5.08	0.31	2.47	2.39	2.77	2.62	90	5.11	5.37	4.47	5.20	2.50	2.39	2.83	2.67
S7	99	0.70	4.62	4.63	3.56	4.83	0.46	2.57	2.55	2.99	2.74	69	5.38	5.30	4.14	5.23	2.44	2.47	3.17	2.77
S8	102	0.88	5.24	5.73	4.32	5.74	0.32	3.20	2.94	3.36	3.20	90	5.41	5.91	4.44	5.96	3.14	2.85	3.46	3.12
S9	107	0.90	6.65	6.74	3.21	6.57	0.30	2.43	2.35	2.46	2.70	96	6.74	6.91	3.18	6.76	2.41	2.28	2.47	2.66
S10	99	0.78	5.80	5.75	2.78	5.86	0.42	2.67	2.71	2.37	2.78	77	6.47	6.44	2.95	6.44	2.34	2.34	2.40	2.62
S11	101	0.87	5.48	5.68	4.71	6.18	0.33	2.87	2.74	3.07	2.71	88	5.55	5.90	4.67	6.33	2.87	2.71	3.05	2.70
S12	99	0.97	6.33	6.69	4.67	6.29	0.17	2.77	2.63	3.15	3.04	96	6.49	6.82	4.65	6.42	2.67	2.55	3.16	3.00
S13	101	0.94	4.54	4.93	4.94	4.92	0.24	2.70	2.61	3.11	2.95	95	4.62	4.91	5.03	4.84	2.75	2.62	3.16	3.00
S14	99	0.91	5.68	5.56	4.35	5.39	0.29	2.62	2.51	2.98	2.70	90	5.76	5.61	4.38	5.44	2.62	2.54	3.01	2.70
S15	102	0.97	4.42	4.96	5.03	5.25	0.17	2.68	2.67	3.13	2.69	99	4.41	4.96	5.15	5.24	2.64	2.63	3.09	2.66
S16	107	0.93	5.25	5.54	4.62	5.64	0.26	2.73	2.58	3.03	2.90	99	5.20	5.63	4.54	5.69	2.79	2.62	3.09	2.96
S17	101	0.93	4.17	4.65	7.01	4.70	0.25	2.99	2.92	3.10	3.26	94	4.17	4.77	7.23	4.80	3.00	2.93	2.96	3.30
S18	101	0.85	5.55	5.40	4.17	5.93	0.36	2.73	2.72	3.05	3.03	86	5.72	5.65	4.27	6.13	2.70	2.63	3.04	2.89
S19	99	0.93	5.37	5.70	4.87	5.48	0.26	2.56	2.65	3.01	2.92	92	5.46	5.73	5.04	5.60	2.59	2.63	3.01	2.92
S20	101	0.91	4.85	5.01	5.05	5.20	0.28	2.44	2.40	3.19	2.53	92	4.89	5.12	5.32	5.16	2.51	2.43	3.20	2.50
S21	99	0.92	4.77	5.15	5.56	4.68	0.27	2.93	2.90	3.29	2.99	91	4.77	5.14	5.70	4.65	2.95	2.93	3.32	3.05
S22	102	0.88	6.46	6.87	3.65	6.45	0.32	2.81	2.62	2.84	3.00	90	6.67	7.13	3.80	6.56	2.76	2.50	2.92	2.94
S23	99	0.92	3.95	4.30	4.95	4.21	0.27	2.60	2.54	3.21	2.86	91	3.89	4.23	5.04	4.14	2.64	2.52	3.20	2.88
S25	99	0.94	5.12	5.49	5.36	5.19	0.24	2.92	2.62	3.19	2.83	93	5.08	5.42	5.47	5.17	2.91	2.54	3.22	2.83
S26	101	0.72	4.67	4.61	3.53	4.94	0.45	2.54	2.56	2.60	2.75	73	5.03	5.19	3.88	5.40	2.52	2.49	2.78	2.70
S27	99	0.94	5.69	5.70	4.26	6.08	0.24	2.83	2.83	3.06	2.95	93	5.83	5.87	4.40	6.16	2.80	2.79	3.10	2.97
S28	101	0.84	3.87	4.11	5.18	4.48	0.37	2.38	2.50	3.00	2.63	85	3.93	4.20	5.56	4.64	2.50	2.62	3.01	2.73
S29	99	0.95	5.30	5.45	4.97	5.22	0.22	2.66	2.60	3.20	2.78	94	5.23	5.45	4.91	5.20	2.65	2.56	3.24	2.80
S30	102	0.76	5.03	4.76	3.97	4.97	0.42	2.51	2.56	2.71	2.73	78	5.36	5.29	4.37	5.53	2.56	2.55	2.76	2.66
S31	107	0.85	4.89	4.98	4.15	5.14	0.36	2.81	2.65	2.95	2.94	91	5.26	5.41	4.35	5.52	2.71	2.51	2.99	2.90
S32	99	0.95	6.72	6.94	2.94	6.99	0.22	2.79	2.60	2.53	2.70	94	6.84	7.00	2.91	7.06	2.78	2.62	2.58	2.73
S33_Long	95	0.92	5.89	5.93	5.53	5.51	0.28	2.98	2.80	3.06	3.06	87	5.95	6.02	5.76	5.59	3.01	2.80	3.06	3.10
S33_Short	99	0.88	4.41	4.16	5.44	4.55	0.33	2.73	2.44	2.94	2.70	87	4.34	4.28	5.64	4.56	2.77	2.53	2.90	2.81
H1	101	0.53	4.41	4.36	3.44	4.47	0.50	2.79	2.80	2.83	2.86	54	5.74	5.65	4.19	5.78	2.64	2.64	2.84	2.78
H2	99	0.91	4.34	4.80	5.34	4.62	0.29	2.70	2.69	3.19	2.81	90	4.37	4.92	5.37	4.66	2.73	2.67	3.19	2.80
H3	102	0.89	5.44	5.73	4.39	5.69	0.31	2.46	2.41	2.79	2.65	91	5.45	5.74	4.32	5.75	2.53	2.44	2.82	2.68
H4_Long	95	0.92	7.08	7.32	3.31	6.94	0.28	2.59	2.40	2.76	2.65	87	7.14	7.41	3.18	7.07	2.68	2.45	2.78	2.71
H4_Short	99	0.85	5.02	5.47	3.97	5.28	0.36	2.63	2.55	2.84	2.84	84	5.21	5.65	3.99	5.43	2.61	2.54	2.84	2.83
H5	107	0.95	5.43	5.66	4.18	5.35	0.21	2.57	2.57	2.93	2.77	102	5.50	5.74	4.27	5.30	2.55	2.52	2.94	2.76
H6	99	0.92	5.73	5.31	4.00	5.55	0.27	2.71	2.59	2.89	2.79	91	5.75	5.30	4.09	5.57	2.59	2.49	2.88	2.76
H7	101	0.85	6.39	6.35	3.54	6.47	0.36	2.32	2.39	2.64	2.76	86	6.70	6.64	3.63	6.71	1.99	2.10	2.62	2.50
H8	99	0.89	5.88	5.90	4.59	5.75	0.31	2.69	2.78	3.12	3.03	88	6.06	6.17	4.75	5.90	2.74	2.74	3.22	3.07
H9	101	0.87	5.29	5.20	3.69	5.45	0.33	2.74	2.71	2.55	2.80	88	5.32	5.18	3.57	5.30	2.72	2.75	2.51	2.76
H10	99	0.91	6.22	6.72	3.35	6.58	0.29	2.60	2.33	2.89	2.57	90	6.16	6.71	3.19	6.50	2.58	2.28	2.82	2.56
H11	102	0.89	5.97	6.26	3.52	6.29	0.31	2.50	2.40	2.63	2.69	91	6.19	6.43	3.60	6.47	2.41	2.32	2.63	2.61
H12	107	0.84	4.79	4.80	4.18	5.03	0.37	2.65	2.50	2.89	2.80	90	5.03	5.13	4.34	5.27	2.58	2.41	2.89	2.71
H13	99	0.86	6.48	6.00	3.00	6.14	0.35	2.64	2.74	2.57	2.95	85	6.52	6.13	3.21	6.27	2.56	2.66	2.68	2.86
H14	102	0.93	5.42	5.76	3.92	5.90	0.25	2.75	2.75	2.85	2.84	95	5.54	5.93	3.95	6.01	2.75	2.67	2.81	2.74
H15	99	0.77	4.42	4.38	3.95	4.92	0.42	2.72	2.81	3.03	3.01	76	4.88	4.86	4.32	5.36	2.65	2.72	3.05	2.89
H16	101	0.91	6.41	6.81	3.89	6.53	0.28	2.62	2.64	2.80	2.84	92	6.51	6.90	3.95	6.59	2.66	2.66	2.85	2.83
H17	99	0.93	5.29	5.80	5.12	5.60	0.26	2.94	2.86	3.25	3.10	92	5.43	5.91	5.10	5.72	2.95	2.88	3.27	3.14
H18	102	0.84	6.41	6.40	3.71	6.30	0.36	2.83	2.59	2.78	2.84	86	6.73	6.76	3.86	6.66	2.62	2.26	2.85	2.61
H19	107	0.93	6.24	6.33	4.78	5.90	0.26	2.86	2.59	3.06	2.98	99	6.30	6.37	4.79	5.91	2.82	2.56	3.04	2.99
H20	99	0.94	4.95	5.22	4.25	5.18	0.24	2.82	2.50	3.13	2.80	93	4.91	5.26	4.29	5.20	2.85	2.52	3.18	2.84
H21	101	0.87	5.57	5.80	3.64	5.78	0.33	3.01	3.19	2.82	2.97	88	5.83	6.05	3.67	5.95	2.94	3.09	2.87	2.89
H22	99	0.96	6.25	6.04	3.26	6.63	0.20	2.63	2.79	2.70	2.73	95	6.26	6.07	3.25	6.66	2.68	2.78	2.73	2.68
H24	101	0.86	5.79	6.01	3.80	6.14	0.35	2.80	2.90	2.98	3.05	87	5.93	6.14	3.80	6.25	2.79	2.86	2.99	3.03
H25	99	0.92	5.02	5.37	5.03	5.30	0.27	2.66	2.72	3.12	2.82	91	5.03	5.38	5.07	5.38	2.65	2.75	3.17	2.87
H26	102	0.85	6.00	6.17	2.82	5.84	0.35	2.59	2.52	2.29	2.70	87	6.40	6.56	2.85	6.18	2.47	2.30	2.31	2.54
H27	101	0.86	5.91	6.01	3.52	6.07	0.35	2.84	2.77	2.76	2.89	87	6.25	6.31	3.78	6.41	2.71	2.55	2.84	2.76
H28	99	0.78	4.79	4.65	3.31	4.90	0.42	2.56	2.58	2.62	2.75	77	5.27	5.29	3.60	5.55	2.48	2.35	2.76	2.61
H29	107	0.90	5.64	5.57	3.47	5.66	0.30	2.49	2.61	2.61	2.88	96	5.73	5.60	3.66	5.70	2.48	2.56	2.67	2.84
H31	99	0.80	5.41	5.23	3.66	5.41	0.40	2.57	2.59	2.67	2.83	79	5.92	5.67	3.80	5.81	2.45	2.46	2.67	2.77
H32	101	0.93	4.32	4.54	6.22	4.52	0.25	2.61	2.55	3.17	2.67	94	4.23	4.39	6.36	4.35	2.57	2.44	3.14	2.61
H33	99	0.90	6.20	6.33	3.43	6.40	0.30	2.66	2.48	2.70	2.58	89	6.37	6.57	3.46	6.54	2.61	2.34	2.78	2.55
H34	102	0.95	5.28	5.78	4.95	5.18	0.22	2.81	2.76	2.90	2.94	97	5.29	5.71	4.99	5.25	2.82	2.68	2.89	2.90
H35	107	0.92	7.25	7.10	2.78	7.18	0.28	2.39	2.27	2.28	2.51	98	7.47	7.31	2.66	7.30	2.29	2.20	2.25	2.47
H36	99	0.78	4.80	4.74	3.53	4.94	0.42	2.77	2.81	2.84	2.96	77	5.22	5.13	3.88	5.31	2.64	2.75	2.88	2.86
H37	101	0.84	5.70	5.69	4.37	6.13	0.37	2.69	2.86	2.82	2.73	85	5.85	6.08	4.54	6.31	2.66	2.76	2.88	2.66
H38	99	0.96	5.27	5.58	5.23	5.73	0.20	3.14	2.97	3.25	3.30	95	5.23	5.57	5.32	5.68	3.13	2.99	3.29	3.36
H39	101	0.94	5.31	5.94	4.56	5.76	0.24	2.41	2.35	2.88	2.74	95	5.33	5.93	4.63	5.75	2.44	2.35	2.91	2.76
H40	99	0.72	4.44	4.28	4.46	4.39	0.45	2.68	2.75	3.14	2.92	71	4.93	4.94	5.10	4.90	2.60	2.71	3.12	2.92
H41	102	0.81	5.66	5.65	3.25	5.77	0.39	2.62	2.59	2.43	2.60	83	5.89	5.92	3.45	6.11	2.49	2.40	2.36	2.40
Trick1	107	0.80	5.14	5.08	3.14	5.50	0.40	2.67	2.69	2.39	3.04	86	5.73	5.62	3.36	5.99	2.48	2.45	2.42	2.91
Trick2	99	0.84	5.54	5.21	2.71	5.43	0.37	2.48	2.65	2.09	2.90	83	5.80	5.57	2.78	5.75	2.33	2.47	2.12	2.84
Trick3	101	0.91	5.77	5.92	3.26	6.24	0.28	2.76	2.62	2.59	2.80	92	5.97	6.05	3.18	6.23	2.72	2.62	2.58	2.78
Trick4	99	0.93	5.82	5.89	4.41	5.61	0.26	3.13	3.11	3.39	3.29	92	6.00	6.04	4.41	5.82	3.15	3.08	3.45	3.30
Trick5	99	0.82	5.77	5.63	4.58	5.62	0.39	2.73	2.74	3.11	3.09	81	6.15	6.16	4.99	5.93	2.77	2.59	3.16	3.19
Trick6_Short	95	0.94	5.49	5.89	4.39	6.13	0.24	2.68	2.41	2.87	2.71	89	5.45	5.89	4.34	6.11	2.71	2.41	2.89	2.71
Trick6_Long	99	0.89	5.95	6.12	4.24	5.90	0.31	2.88	2.76	2.80	3.06	88	5.88	6.13	4.26	5.97	2.95	2.69	2.77	3.01
Trick7_Short	99	0.89	3.80	4.11	5.84	3.96	0.31	2.66	2.71	3.33	2.69	88	3.86	4.16	6.09	3.97	2.69	2.70	3.26	2.69
Trick7_Long	95	0.95	6.14	6.16	4.82	5.94	0.22	2.72	2.75	3.14	2.92	90	6.19	6.22	4.93	5.97	2.75	2.76	3.17	2.95
Trick8_Short	95	0.94	5.56	5.56	3.56	5.74	0.24	2.71	2.72	2.54	2.72	89	5.62	5.65	3.55	5.85	2.75	2.74	2.58	2.74
Trick8_Long	99	0.89	5.47	5.58	3.60	5.29	0.31	2.58	2.55	2.66	2.84	88	5.51	5.69	3.50	5.41	2.59	2.55	2.69	2.83
Trick10	102	0.68	5.83	5.79	2.65	5.83	0.47	2.66	2.73	1.96	2.88	69	6.71	6.55	2.94	6.54	2.20	2.21	2.05	2.49
Trick11	107	0.87	6.79	6.75	2.82	6.60	0.34	2.82	2.94	2.40	3.04	93	7.04	7.09	2.54	6.89	2.71	2.83	2.18	3.01
Trick12_Short	99	0.90	4.52	4.85	4.38	4.78	0.30	2.51	2.37	2.97	2.69	89	4.55	4.93	4.30	4.80	2.58	2.44	3.06	2.76
Trick12_Long	95	0.91	6.29	6.18	3.15	6.25	0.29	2.79	2.79	2.58	2.96	86	6.33	6.21	3.09	6.28	2.76	2.75	2.56	2.97
Trick13	99	0.91	5.45	5.80	3.76	5.83	0.29	2.72	2.61	2.84	2.75	90	5.38	5.82	3.94	5.86	2.74	2.58	2.87	2.70
Trick14_Short	95	0.94	5.24	5.29	3.73	5.61	0.24	2.71	2.68	2.77	2.77	89	5.27	5.27	3.70	5.62	2.72	2.64	2.76	2.75
Trick14_Long	99	0.85	4.93	4.93	4.04	4.88	0.36	2.68	2.66	2.91	2.84	84	4.99	5.06	4.08	5.00	2.66	2.61	2.92	2.79
Trick15	101	0.81	5.32	5.47	3.71	5.64	0.39	2.64	2.65	2.74	2.79	82	5.56	5.87	3.79	5.83	2.65	2.57	2.76	2.76
Trick16_Short	99	0.86	4.70	4.91	3.46	5.15	0.35	2.60	2.60	2.52	2.78	85	4.76	4.98	3.67	5.21	2.56	2.51	2.58	2.69
Trick16_Long	95	0.75	5.94	5.77	3.02	5.89	0.43	2.74	2.86	2.51	2.91	71	6.48	6.39	3.30	6.48	2.59	2.69	2.70	2.76
Trick17	101	0.84	5.45	5.53	3.39	5.77	0.37	2.55	2.57	2.67	2.75	85	5.65	5.73	3.38	5.74	2.60	2.57	2.66	2.78
Trick18_Long	99	0.86	5.02	5.22	3.55	5.27	0.35	2.82	2.75	2.70	3.02	85	5.22	5.56	3.67	5.58	2.83	2.69	2.71	2.96
Trick18_Short	95	0.88	5.32	5.04	3.44	5.44	0.32	2.71	2.53	2.70	2.95	84	5.44	5.21	3.45	5.63	2.71	2.49	2.70	2.95
Trick19	101	0.93	6.57	6.91	3.47	6.65	0.25	2.65	2.45	2.59	2.77	94	6.69	7.05	3.38	6.80	2.61	2.33	2.50	2.76
Trick20_Short	99	0.90	5.71	5.89	4.31	5.51	0.30	2.93	2.76	3.13	3.18	89	5.85	6.01	4.30	5.58	2.89	2.69	3.10	3.17
Trick20_Long	95	0.83	6.18	6.04	3.47	5.87	0.37	2.66	2.79	2.61	2.93	79	6.48	6.54	3.54	6.33	2.62	2.61	2.73	2.85
Trick21	99	0.82	5.23	4.94	3.94	4.75	0.39	2.64	2.82	3.09	2.92	81	5.46	5.22	4.12	4.98	2.64	2.80	3.18	2.91
Trick22_Long	99	0.90	5.97	6.05	4.02	5.86	0.30	2.57	2.65	2.95	2.89	89	6.07	6.12	4.17	5.91	2.52	2.56	3.01	2.85
Trick22_Short	95	0.91	5.47	5.47	4.61	5.49	0.29	2.70	2.47	2.95	2.71	86	5.58	5.62	4.65	5.50	2.70	2.39	2.92	2.77
Trick23_Short	95	0.58	4.76	4.38	3.07	4.68	0.49	2.78	2.77	2.75	2.95	55	5.87	5.75	3.95	5.78	2.62	2.59	2.96	2.96
Trick23_Long	99	0.72	5.70	5.34	2.82	5.33	0.45	2.91	3.19	2.45	3.19	71	6.52	6.10	2.99	6.21	2.59	2.96	2.49	2.95
Trick24	102	0.86	4.17	4.38	3.82	4.61	0.34	2.37	2.33	2.69	2.57	88	4.33	4.63	3.89	4.98	2.39	2.32	2.70	2.54
Trick25	107	0.93	5.97	6.40	3.07	6.42	0.25	2.74	2.63	2.65	2.90	100	6.08	6.56	3.04	6.61	2.70	2.60	2.65	2.83
Trick26	99	0.86	4.26	4.30	3.27	4.46	0.35	2.41	2.29	2.42	2.63	85	4.35	4.39	3.40	4.66	2.39	2.27	2.53	2.64
Trick27	101	0.89	6.82	6.54	2.87	6.49	0.31	2.52	2.68	2.36	2.90	90	7.07	6.77	2.90	6.76	2.36	2.51	2.35	2.73
Trick28	99	0.95	4.59	5.28	4.98	5.23	0.22	2.78	2.56	2.91	2.83	94	4.53	5.28	5.03	5.20	2.83	2.60	2.94	2.88
Trick29	101	0.84	4.98	5.29	4.18	5.09	0.37	2.42	2.61	2.72	2.59	85	5.19	5.60	4.48	5.40	2.33	2.53	2.73	2.55
Trick30	99	0.89	6.58	6.40	3.74	6.41	0.31	2.31	2.42	2.91	2.68	88	6.70	6.49	3.76	6.51	2.17	2.36	2.96	2.59
Trick31	102	0.92	6.06	6.25	3.25	6.15	0.27	2.41	2.46	2.46	2.70	94	6.13	6.37	3.41	6.27	2.34	2.29	2.49	2.61
Trick32_Long	99	0.92	4.12	4.40	5.93	4.16	0.27	2.68	2.61	3.24	2.90	91	3.98	4.34	6.04	4.13	2.61	2.58	3.24	2.91
Trick32_Short	95	0.84	4.59	4.41	4.95	4.85	0.36	2.89	2.70	3.22	3.04	80	4.68	4.56	5.25	5.03	2.91	2.76	3.17	3.14
Trick33	107	0.93	6.62	6.95	3.15	6.91	0.25	2.58	2.43	2.49	2.73	100	6.71	7.02	3.03	7.01	2.57	2.49	2.45	2.73
Trick34	99	0.81	6.44	5.99	2.83	6.01	0.39	2.50	2.56	2.36	2.95	80	6.59	6.14	2.94	6.13	2.45	2.50	2.42	2.98
Trick35	101	0.89	4.26	4.29	4.99	4.53	0.31	2.79	2.61	3.27	2.98	90	4.24	4.41	5.23	4.61	2.82	2.66	3.30	3.02
Trick36_Long	95	0.56	5.48	5.23	2.61	5.37	0.50	2.79	2.92	2.33	2.97	53	6.47	6.38	3.17	6.40	2.60	2.61	2.52	2.70
Trick36_Short	99	0.72	4.98	4.94	3.95	5.02	0.45	2.62	2.55	2.92	2.97	71	5.35	5.52	4.31	5.32	2.76	2.52	2.92	2.95
Trick37	99	0.86	5.81	5.86	3.40	5.70	0.35	2.77	2.70	2.92	2.94	85	6.36	6.42	3.45	6.26	2.51	2.40	2.96	2.74
Trick38	101	0.90	4.37	4.57	4.96	4.56	0.30	2.57	2.49	3.03	2.72	91	4.37	4.58	4.98	4.56	2.68	2.53	3.13	2.80
Trick39	99	0.88	5.23	5.54	3.77	5.43	0.33	2.51	2.39	2.80	2.48	87	5.36	5.79	3.78	5.66	2.59	2.35	2.84	2.43
Trick40	102	0.92	4.97	5.09	5.15	5.26	0.27	2.94	2.62	3.08	2.77	94	4.80	5.11	5.24	5.09	2.89	2.67	3.12	2.70
Trick41	107	0.89	5.57	5.52	4.13	5.58	0.32	2.59	2.51	2.89	2.86	95	5.71	5.66	4.26	5.76	2.66	2.52	2.90	2.92

**Table 8:** Between-person correlations among ratings

	1	2	3
1: surprise in response to the trick			
2: interest in the trick	0.91*		
3: confidence in the solution	– 0.22***	– 0.09*	
4: curiosity in the solution	0.84***	0.89***	– 0.12*

**p* < .05, ** *p* < .01, *** *p* < .001

## Footnotes

1 A limited number of studies used different materials to elicit epistemic emotions such as blurred pictures (Jepma, Verdonschot, van Steenbergen, Rombouts, & Nieuwenhuis, 2012), visual arts (Silvia, 2005), and philosophical quotations (Fayn, Silvia, Dejonckheere, Verdonck, & Kuppens, 2019).
